# Transhepatic Hilar Approach for Perihilar Cholangiocarcinoma: Significance of Early Judgment of Resectability and Safe Vascular Reconstruction

**DOI:** 10.1007/s11605-016-3332-7

**Published:** 2016-11-28

**Authors:** Naohisa Kuriyama, Shuji Isaji, Akihiro Tanemura, Yusuke Iizawa, Hiroyuki Kato, Yasuhiro Murata, Yoshinori Azumi, Masashi Kishiwada, Shugo Mizuno, Masanobu Usui, Hiroyuki Sakurai

**Affiliations:** Department of Hepatobiliary Pancreatic and Transplant Surgery, Mie University Graduate School of Medicine, 2-174 Edobashi, Tsu City, Mie 514-8507 Japan

**Keywords:** Perihilar cholangiocarcinoma, Transhepatic hilar approach, Portal vein resection

## Abstract

In the most common surgical procedure for perihilar cholangiocarcinoma, the margin status of the proximal bile duct is determined at the final step. Our procedure, the transhepatic hilar approach, confirms a cancer-negative margin status of the proximal bile duct first. We first performed a partial hepatic parenchymal transection to expose the hilar plate, and then transected the proximal bile duct to confirm margin status. Then, divisions of the hepatic artery and portal vein of the future resected liver are performed, followed by the residual hepatic parenchymal transection. The transhepatic hilar approach offers a wide surgical field for safe resection and reconstruction of the portal vein in the middle of the hepatectomy. We reviewed 23 patients with perihilar cholangiocarcinoma who underwent major hepatectomy using our procedure from 2011 to 2015. A combined vascular resection and reconstruction was carried out in 14 patients (60.9%). R0 resection was achieved in 17 patients (73.9%), and the overall 3-year survival rate was 52.9% (median survival time 52.4 months). The transhepatic hilar approach is useful and practicable regardless of local tumor extension, enabling us to determine tumor resectability and perform safe resection and reconstruction of the portal vein early in the operation.

## Introduction

Surgery for perihilar cholangiocarcinoma demands precise preoperative evaluation and management by very skilled hepatobiliary surgeons. In the past two decades, advances in diagnostic and surgical techniques have improved surgical outcomes and survival rates.^1^ R0 resection is an important factor for achieving a good prognosis for perihilar cholangiocarcinoma.^2^ This demands not only a major hepatectomy with caudate lobectomy but also suitable skeletonization and treatment of the hepatic artery, portal vein, and bile duct individually. Bismuth described two basic procedures for a typical hepatectomy. One consisted of hepatectomy with preliminary vascular control, first performed by Honjo of Kyoto University in 1949. The other was hepatectomy by primary parenchymatous transection, which began with the opening of the parenchyma along the line of the scissura described by Ton That Tung.^3^ Using the same concept as primary parenchymatous transection, Miyazaki et al. recently reported the usefulness of the transhepatic approach for hilar cholangiocarcinoma, in which extensive hilar bile duct resection was performed without excision of any liver parenchyma. This was appropriate for patients with liver dysfunction for whom major hepatectomy was contraindicated.^4^ This approach offers a sufficient surgical view to visualize the hilar bile duct and an easy approach to the portal vein and hepatic artery after partial parenchymal division. In perihilar cholangiocarcinoma, the critical aspect for curative resection is the cut margin of the remnant liver, including the hepatic artery, portal vein, and bile duct. It is preferable to determine the resectability and possibility of reconstruction of the portal vein and/or hepatic artery early in the operation. To achieve this, in early 2011, we modified a primary hepatic parenchymal transection technique and developed a new operative procedure with major hepatectomy for perihilar cholangiocarcinoma, called the transhepatic hilar approach (THA). Here, we report our experience using the THA technique and our associated results.

## Materials and Methods

Between January 2011 and December 2015, 23 patients with perihilar cholangiocarcinoma underwent major hepatic resection with caudate lobectomy using the THA followed by hepatectomy with curative intent at our institution. The patients consisted of 13 men and 10 women, with an average age of 70 years (range 51–87 years). Multidetector-row computed tomography (MDCT), endoscopic retrograde cholangiography (ERC), and intraductal ultrasonography (IDUS) were used in all patients for preoperative tumor staging. Tumor and negative biopsies by ERC were used for confirming diagnosis and definition of biliary cancer invasion. Endoscopic retrograde biliary drainage (ERBD) tubes were inserted into the future remnant liver in all patients with obstructive jaundice.

After evaluation of tumor extension into the hepatic artery, portal vein, and bile duct by preoperative imaging studies, two cycles of chemotherapy with gemcitabine (600 mg/m^2^ on days 7 and 21) plus S-1 (60 mg/m^2^ daily on days 1–21 every 4 weeks),^5^^,^ 6 followed by surgery, was administered to the 16 patients with local advanced perihilar cholangiocarcinoma with (1) main, bilateral, or contralateral portal vein and/or hepatic artery invasion with or without possible vascular reconstruction; or (2) invasion of the right side of the umbilical portion (U portion) and the left side of the origin of the right posterior portal vein (P portion); or (3) regional lymph node metastasis. Of these, two patients received additional chemoradiotherapy because it was determined that curative intent resection was impossible after completion of chemotherapy. The total radiation dose for these two patients was 36 Gy delivered in daily fractions of 1.8 Gy five times per week. One of the patients was administered three infusions of gemcitabine (800 mg/body) and the other was administered four cycles of gemcitabine (1000 mg/body) plus cisplatin (25 mg/body) on days 1 and 8 intravenously every 3 weeks.^7^

Based on preoperative imaging, we determined on which side the hepatectomy should be performed. Right hepatectomy was applied to Bismuth type I, II, and IIIa tumors. Left hepatectomy was applied to Bismuth type IIIb tumors. If a tumor obviously extended over the second order biliary radicles, such as Bismuth type IV tumors, trisectionectomy or central bisectionectomy was selected. Combined with the abovementioned anatomical criteria for hepatectomy, the type of hepatectomy was determined using the following factors: the indocyanine green retention rate at 15 min (ICGR15), the hepatic uptake ratio of 99mTc-GSA scintigraphy at 15 min (LHL15), and the future remnant liver volume using computed tomography (CT) volumetry.^8^ Portal vein embolization (PVE) was indicated when the future remnant liver volume was estimated as less than 40%.

For the patients with Bismuth type I and II perihilar cholangiocarcinoma, limited extrahepatic bile duct resection without hepatectomy is occasionally performed, but our institution usually employs right hepatectomy with caudate lobectomy, based on previous studies. Ikeyama et al. performed a retrospective study on 31 consecutive patients who underwent resection of these types of tumors.^9^ R0 resection and survival rates of patients who underwent right hepatectomy with caudate lobectomy (*n* = 18) were significantly better than those of patients who underwent other types of resection (*n* = 13). In that study, most patients did not have invasion of the right hepatic artery, but the distance between the leading edge of the cancer and the outer layer of the hepatic artery was 1 mm in many patients. The authors suggested that the resected margin would have been cancer positive without combined resection of the right hepatic artery. Therefore, they recommend right hepatectomy even when invasion of the right hepatic artery cannot be demonstrated preoperatively by diagnostic imaging. Two additional small studies also reported that outcomes of limited resection except for those of right hepatectomy with caudate lobectomy were unsatisfactory with low curative resection rate and survival rate.^10^^,^ 11 Further evaluation with larger sample sizes is required to justify right hepatectomy with caudate lobectomy for Bismuth type I and II perihilar cholangiocarcinoma.

### Concept of THA for Perihilar Cholangiocarcinoma

The most common procedure for perihilar cholangiocarcinoma previously reported by Japanese surgeons is to first resect the common bile duct above the pancreas and skeletonize the hepatoduodenal ligament, followed by division of the hepatic artery and portal vein of the future resected liver, hepatic parenchymal transection along the demarcation line, and finally transection of the hepatic bile duct of the future remnant liver.^12^^,^ 13 In this procedure, the cut margin of the hepatic bile duct is examined in the final stage.

Our THA procedure is very different from the previously reported one in terms of when the hepatic bile duct transection is performed. Our policy is to initially confirm a cancer-negative margin of the hepatic bile duct. In THA, we first performed partial hepatic parenchymal transection toward the hepatic hilum to expose the hilar plate, followed by exposure of the hepatic artery, portal vein, and bile duct in the future remnant liver. Then, transection of the hepatic bile duct is performed to confirm a cancer-negative margin, followed by resection of the common bile duct above the pancreas and skeletonization of the hepatoduodenal ligament. Then, division of the hepatic artery and portal vein of the future resected liver is performed before transection of the residual hepatic parenchyma.

Partial hepatic parenchymal transection using THA gives a better surgical view of the cranial side of the hilar plate and provides an improved surgical field for safe encircling and taping of the proximal bile duct, portal vein, and hepatic artery compared with performing these procedures without hepatic parenchymal transection.

### Surgical Procedures of THA for Perihilar Cholangiocarcinoma

Under an inverted-T incision, careful exploration of peritoneal dissemination and liver metastasis is performed. If there are no obvious unresectable factors, taping of the hepatoduodenal ligament using a Penrose drain and of the intrahepatic inferior vena cava using sailor tape is first performed for the control of accidental bleeding, and hepatic mobilization is started. After dividing the bilateral coronary and triangular ligaments, the short hepatic veins are divided to mobilize the caudate lobe from the IVC.

The line of the middle hepatic vein (MHV) is marked on the liver surface using electric cautery under ultrasonographic (US) guidance, and the MHV is preserved on the side of the future remnant liver. Following detachment of the gallbladder from the gallbladder bed, the hepatic parenchyma transection is started from the caudal-ventral edge of the Rex-Cantlie line and advanced in the cranio-dorsal direction by using a Cavitron ultrasonic surgical aspirator (CUSA) and monopolar electrode irrigated with saline (vessel sealer device) under the Pringle maneuver using a 15-min clamp and 5-min declamp. When we encountered troublesome bleeding without formal vascular control during THA procedure, we generally pressed the bleeding site by gauze or grasp the bleeding vessel by the forceps, and then performed Pringle maneuver to control bleeding, followed by hemostasis using vessel sealing device and/or suturing technique. The advancement of surgical device and tissue sealing sheet enabled us to achieve enough hemostasis at the hepatic cut surface.

**Fig. 1:**
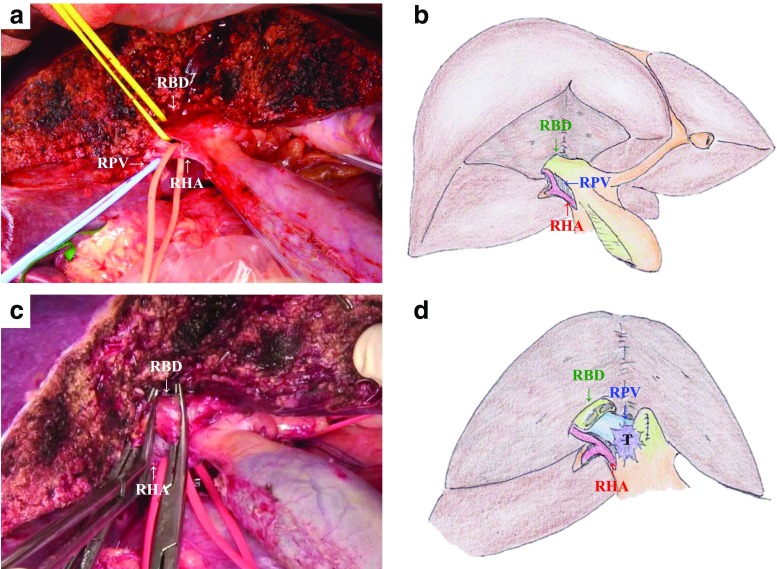
Transhepatic hilar approach of left hepatectomy for perihilar cholangiocarcinoma. **a**, **b** The hepatic transection progresses to the root of the anterior glissonial sheath. After completely exposing the planed cutting glisson sheath, the hepatic artery, portal branch, and plate with proximal bile duct are carefully divided and taped at the transected plane. **c**, **d** Dissection of sheath with proximal bile duct is first performed and the frozen sections of the hepatic bile ducts are immediately examined to confirm a cancer-negative margin of the proximal bile ducts. *RHA* right hepatic artery, *RPV* right portal vein, *RBD* right bile duct

**Fig. 2:**
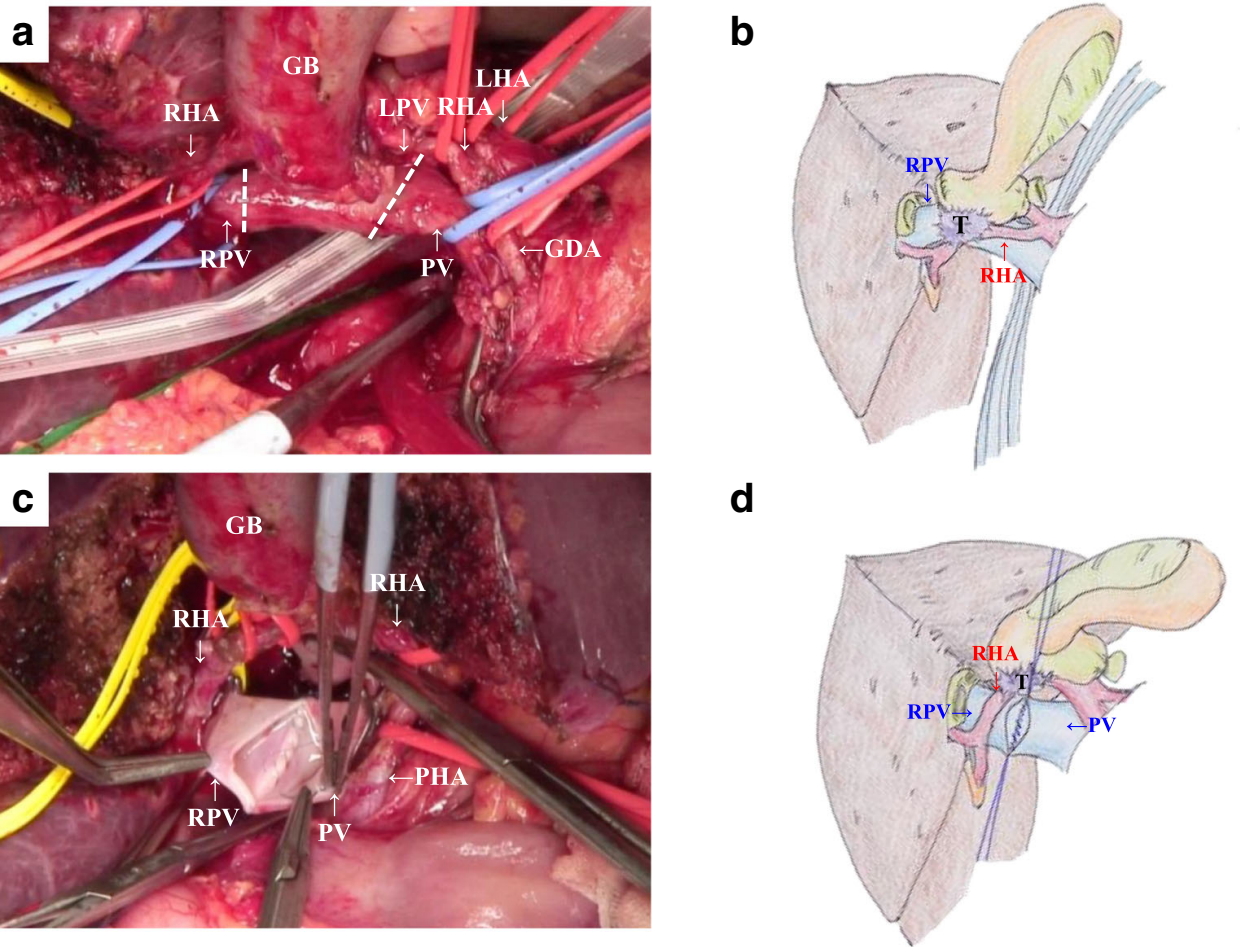
Portal vein resection and reconstruction in the middle of left hepatectomy. **a**, **b** Tumor invaded from left to main portal vein. **c**, **d** Right portal vein resection and reconstruction was easily performed by using the intraluminal suturing technique for the posterior wall and the over-and-over method for the anterior wall using 6-0 vascular sutures under the open and wide surgical view after partial hepatectomy. *T* tumor, *RHA* right hepatic artery, *LHA* left hepatic artery, *PHA* proper hepatic artery, *GDA* gastroduodenal artery, *PV* portal vein, *RPV* right portal vein, *LPV* left portal vein, *GB* gallbladder

**Fig. 3:**
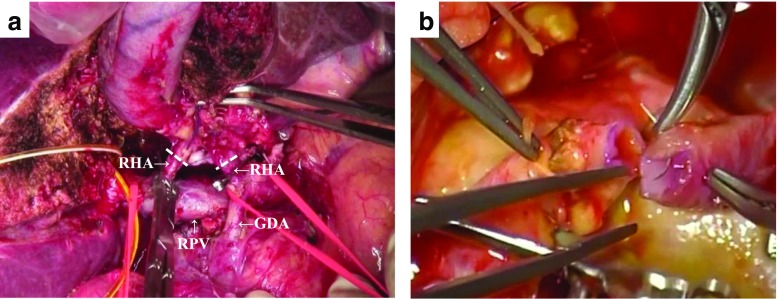
Hepatic artery resection and reconstruction after hepatectomy. **a** Tumor invaded to right hepatic artery (*RHA*). **b** RHA resection and reconstruction was performed under a microscope after completed liver resection with caudate lobe. *RHA* right hepatic artery, *GDA* gastroduodenal artery, *RPV* right portal vein

For left hepatectomy, the left-side line of the MHV is marked on the ventral liver surface. A straight line is also marked on the dorsal liver surface from the caudal-ventral edge of the Rex-Cantlie line to the root of the anterior glissonial pedicle. After exposing the right-side hilar plate, the right hepatic artery, portal branch, and hepatic bile duct are carefully skeletonized and taped at the transected plane (Fig. 1a, b). The shape of the partial hepatic parenchymal transection plane is an isosceles triangle. Resection of the right proximal hepatic bile duct is performed first. In many cases, the right anterior and posterior sections of the bile duct are resected separately. Frozen sections of the hepatic bile ducts are immediately examined to confirm a cancer-negative margin of the proximal bile ducts (Fig. 1c, d). Thereafter, the regional lymph nodes are resected along the common hepatic artery and within the hepatoduodenal ligament while exposing and taping the hepatic arteries, portal trunk, and common bile duct. The common bile duct is transected above the pancreas. The gallbladder and extrahepatic bile duct are reflected in a cranio-ventral direction, and isolation of the portal trunk and the proper hepatic artery is advanced up to the hepatic hilum. After transecting the left hepatic artery and portal vein, the right hepatic artery and portal vein are skeletonized to the bifurcation of the right anterior and posterior sections of the hepatic arteries and portal veins. When there is tumor involvement of the portal vein in the future remnant side (Fig. 2a, b), combined resection and reconstruction of the portal vein can be performed under the wide surgical view obtained by the THA. End-to-end anastomosis is performed by the intraluminal suturing technique for the posterior wall and the over-and-over method for the anterior wall using 6-0 vascular sutures (Fig. 2c, d). At this point, the tumor is completely separated from the residual liver, and the residual hepatic parenchymal transection including the caudate lobe is performed. When the hepatic artery in the future remnant side is also involved, combined resection is performed and its end-to-end anastomosis is performed with 9–0 vascular sutures under a microscope after completion of the left hepatectomy with caudate lobectomy (Fig. 3a, b).

**Fig. 4:**
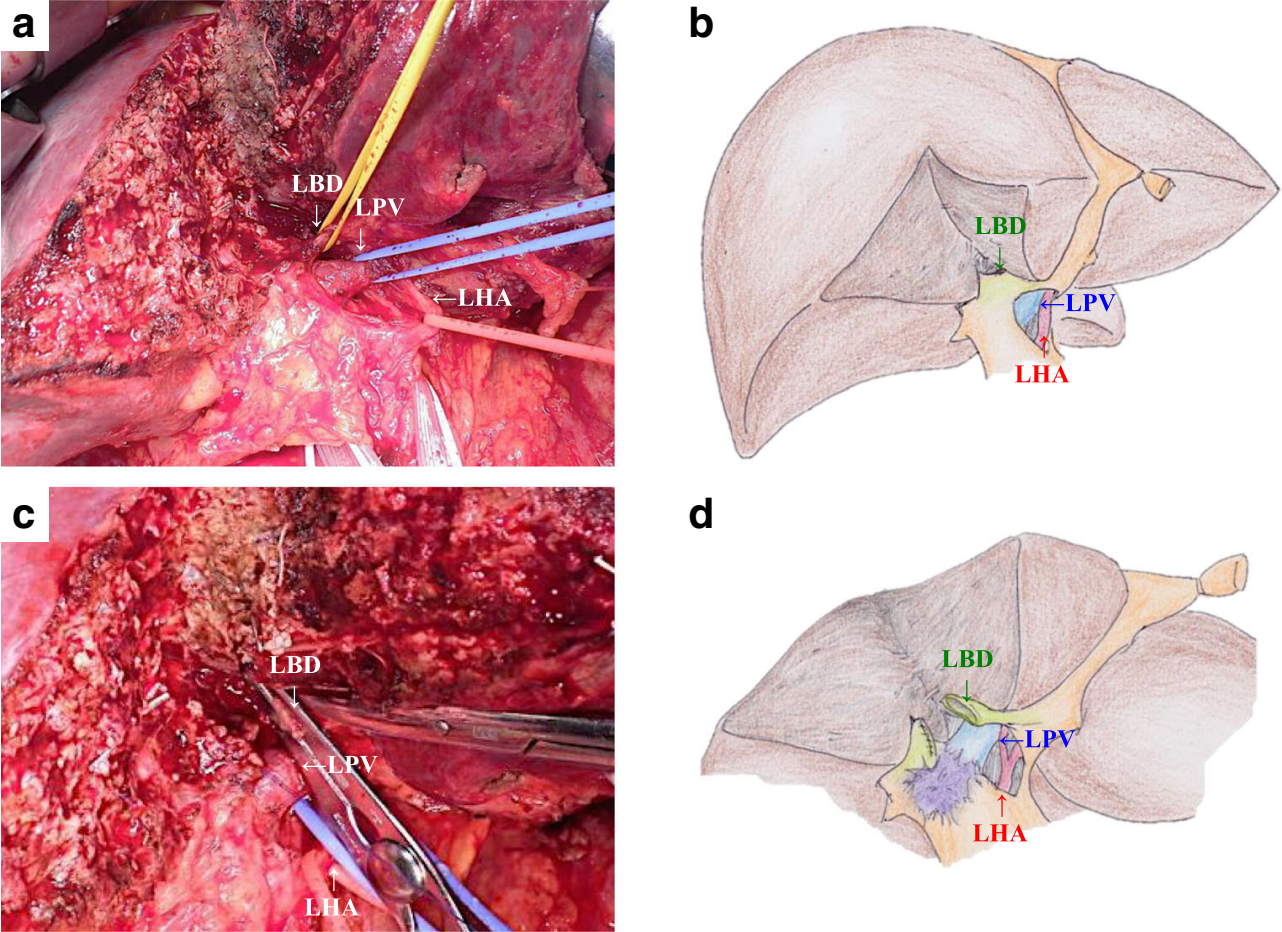
Transhepatic hilar approach of right hepatectomy for perihilar cholangiocarcinoma. **a**, **b** The hepatic transection progresses to the root of the umbilical plate. After completely exposing the planned cutting glisson sheath, the hepatic artery, portal branch, and plate with hepatic bile duct are carefully divided and taped at the transected plane. **c**, **d** Dissection of sheath with proximal bile duct is first performed and the frozen sections of the hepatic bile ducts are immediately examined to confirm a cancer-negative margin of the proximal bile ducts. *LHA* left hepatic artery, *LPV* left portal vein, *LBD* left bile duct

For right hepatectomy, the right-side line of the MHV is marked on the ventral liver surface. A straight line is also marked on the dorsal liver surface from the caudal-ventral edge of the Rex-Cantlie line to the root of the umbilical plate. After exposing the left-side hilar plate, the left hepatic artery, portal branch, and hepatic bile duct are carefully skeletonized and taped at the transected plane (Fig. 4a, b). Resection of the left hepatic bile duct is performed first. In many cases, the left internal sectional bile duct and external sectional bile duct are resected separately. A frozen section of the hepatic bile duct is immediately examined to confirm a cancer-negative margin of the proximal bile duct (Fig. 4c, d). Thereafter, the regional lymph nodes are resected and the hepatic arteries, portal trunk, and common bile duct are exposed and taped. The common bile duct is transected above the pancreas. After transecting the right hepatic artery and portal vein, the left hepatic artery (and middle hepatic artery) and portal vein and left hepatic artery are skeletonized to the right side of the umbilical plate. When there is tumor involvement of the portal vein in the future remnant side, combined resection and reconstruction of the portal vein can be performed. At this point, the tumor is completely separated from the residual liver, and the residual hepatic parenchymal transection including the caudate lobe is performed. When the hepatic artery in the future remnant side is also involved, combined resection is performed as is its end-to-end anastomosis.

The indications for portal vein and hepatic artery resection include the following: (1) to enable taping of the proximal portal vein and hepatic artery in the future remnant liver, (2) to enable sufficient length of the proximal portal vein and hepatic artery to perform end-to-end anastomosis, and (3) to enable R0 resection. If necessary, the right external iliac vein is used as a portal vein graft and several arteries are used for hepatic artery reconstruction.

When performing left hepatectomy, we occasionally encounter three bile duct stumps at the transection plate: the right anterior inferior segmental bile duct (B5), right anterior superior segmental bile duct (B8), and posterior sectional bile duct. During right hepatectomy, there are also two (or three) bile duct orifices at the transected plate: the internal sectional bile duct (B4) and external sectional bile duct (B2 + B3) (or independently B4, B2, B3). In these cases, hepaticojejunostomy with biliary stents in an antecolic Roux-en-Y fashion is performed using either interrupted or continuous sutures with 6-0 absorbable monofilament threads. Especially when the bile duct orifices are very small (less than 5 mm), 12 stitches are sutured on the small anastomosis using the Pair-Watch suturing technique.^14^

## Results

**Fig. 5:**
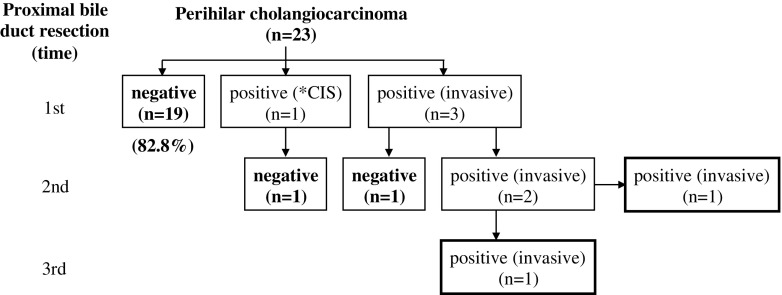
Flow diagram of residual tumor status of proximal bile duct margin according to the frequency of bile duct resection. *CIS carcinoma in situ

**Fig. 6:**
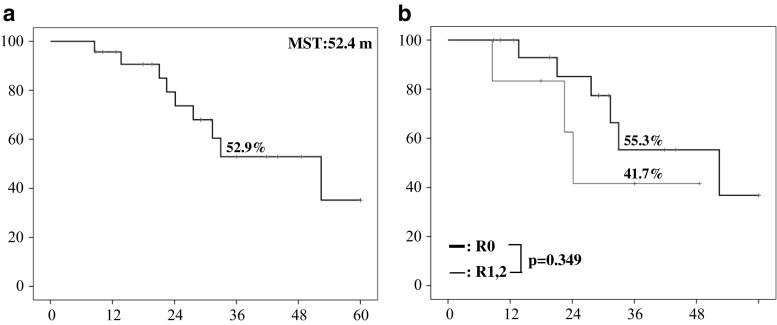
**a**, **b** Disease-specific survival curve of all 23 patients

Neoadjuvant chemotherapy based on GS therapy was performed in 16 (69.6%) of the 23 patients (Table 1). In addition, combined radiation therapy was also performed in two (8.7%) patients. Hepatic resections were performed without any intraoperative complications. Several kinds of hepatectomy with caudate lobectomy and extrahepatic bile duct resection were performed as shown in Table 1: left hepatectomy in 12 patients (52.2%), left trisectionectomy in two patients (8.7%), right hepatectomy in seven patients (30.5%), right trisectionectomy in one patient (4.3%), and central bisectionectomy in one patient (4.3%). A combined vascular resection with reconstruction was performed in 14 patients (60.9%), including the portal vein alone in 11 patients and both portal vein and hepatic artery in three patients. In addition, combined pancreaticoduodenectomy was also performed in one patient. Median operation time was 600 min (range 382–728 min) and median blood loss was 1789 ml (range 556–4978 ml). In the TNM staging system by the UICC (7th edition), pT stage was identified in pT1 in one patient (4.3%), pT2a in four patients (17.4%), pT2b in six patients (26.1%), pT3 in 10 patients (43.5%), and pT4 in two patients (8.7%). Twelve patients (52.5%) had regional lymph node metastasis (pN1) and two patients (8.7%) had intrahepatic metastases (M1). The final tumor stage was stage II in five patients (21.7%), IIIA in six patients (26.1%), IIIB in 10 patients (43.5%), and IVB in two patients (8.7%). R0 resections were performed in 17 (74.0%) of the 23, including two patients with distant metastases. As shown in Table 2, R1 margins were found in three patients (13.0%): the proximal bile duct margin (HM) was positive in one patient and the dissected margin (EM) was positive in two. R2 margins were found in three patients (13.0%): HM was positive in one and intrahepatic metastasis was found in two. Regarding the frequency of bile duct resection required to achieve margin-negative status of the HM (Fig. 5), 19 patients (82.7%) obtained margin-negative status at the first bile duct resection. Carcinoma in situ was found in one patient and invasive carcinoma in the other three. Among these four patients, two patients (8.7%) finally obtained margin-negative status after an additional bile duct resection. Therefore, 21 patients (91.3%) ultimately obtained margin-negative status of the proximal bile duct. However, of these, two patients were postoperatively diagnosed as dissected margin (EM) positive and two patients also had intrahepatic metastasis in the resected specimen. Therefore, 17 patients (74%) actually achieved R0 resection (Table 2). When relating margin-negative status of HM to Bismuth classification, the frequency of margin-negative status at the first bile duct resection was 4/4 (100%) in type IIIa patients, 6/7 (85.7%) in type IIIb patients, and 9/12 (75.0%) in type IV patients. The final frequency of margin-negative status was also 4/4 (100%) in type IIIa patients, 7/7 (100%) in type IIIb patients, and 10/12 (83.3%) in type IV patients. In terms of the postoperative course and prognosis of patients with R1 or R2 resection, two patients who were diagnosed as EM1 positive after major hepatectomy with caudate lobectomy are alive without recurrence at 43 and 28 months after operation, respectively. Of the two patients who were diagnosed with intrahepatic metastasis in the resected specimen after left hepatectomy with caudate lobectomy, one patient died at 9 months and the other patient is alive at 18 months after the operation. Of the two patients who were intraoperatively diagnosed as HM positive, one was HM1 positive (invasive carcinoma) in both the B2 and B3 bile ducts even after additional resection and underwent right trisectionectomy with caudate lobectomy, because PVE was preoperatively performed. This patient developed anastomotic stenosis of the hepaticojejunostomy at 6 months and died at 16 months after operation. The other patient was HM2 positive (invasive carcinoma) in both anterior and posterior sectional bile ducts even after additional resection and underwent left hepatectomy with caudate lobectomy, because the preoperatively estimated hepatic resection rate was less than 30%. This patient developed anastomotic stenosis of the hepaticojejunostomy at 3 months and died at 28 months after the operation. Clavien III or higher postoperative complications occurred in 11 patients (45.8%) (Table 3). Among them, five developed intra-abdominal abscesses due to bile leakage that required percutaneous drainage. Two of these patients also developed ileus that required surgical treatment, followed by liver failure due to the abscesses. Outflow blockage requiring stent insertion at POD8, portal vein thrombosis following portal vein resection and reconstruction requiring stent insertion at POD1, and drainage of pleural effusion each occurred in one patient. The median postoperative hospital stay was 44 days (range 22–136 days). The disease-specific 3-year survival rate was 52.9% (median survival time 52.4 months) (Fig. 6a). R status distribution (Fig. 6b) was 55.3% R0 and 41.7% R1/2 with no statistically significant difference (*p* = 0.349).

**Table 1:** Patients’ characteristics and clinicopathological features

Average age (years)	70 (51–87)
Gender (male/female)	13/10
Preoperative treatment	16 (69.6%)
Chemotherapy	14
Chemoradiotherapy	2
Preoperative portal vein embolization	4 (16.7%)
Bismuth classification	
I/II/IIIa/IIIb/IV	0/0/2/7/14
Type of hepatectomy	
S1, 2, 3, 4/S1, 2, 3, 4, 5, 8	12 (52.2%)/2 (8.7%)
S1, 5, 6, 7, 8/S1, 4, 5, 6, 7, 8	7 (30.5%)/1 (4.3%)
S1, 4, 5, 8	1 (4.3%)
Combined vascular resection	14 (60.9%)
Portal vein alone	11
Portal vein and hepatic artery	3
Pancreatoduodenectomy	1
Operation time: median (range)	556 (383–728) min
Blood loss: median (range)	2029 (556–4978) ml
UICC T stage	
pT1/2a/2b/3/4	1/4/6/10/2
UICC N stage	
pN1	12 (52.2%)
UICC M stage	
M1 (intrahepatic metastases)	2 (8.7%)
UICC final stage	
II/IIIA/IIIB/IVB	5/6/10/2
Residual tumor (R)	
R0/R1/R2	17 (74.0%)/3/3
Postoperative hospital stay: median (range)	44 (22–136) days

*R0* complete resection, *R1* microscopic residual tumor resection, *R2* macroscopic residual tumor resection or distant metastasis

**Table 2:** Residual tumor factors

Residual tumor	Local factor			Distant factor	Total
	pHM(+)	pDM(+)	pEM(+)	pM1	
R1	1	0	2	0	3 (13.0%)
R2	1	0	0	2 (IM)	3 (13.0%)
					6 (26.0%)

*pHM* proximal bile duct margin, *pDM* distal bile duct margin, *pEM* dissected margin, *IM* intrahepatic metastasis

**Table 3:** Complication and in-hospital mortality

Complication (Clavian III or higher)	
Bile leakage	5
Ileus	2
Liver failure	1
Outflow block	1
Portal vein thrombosis	1
Pleural effusion	1
	11 (47.8%)
In-hospital mortality	0

## Discussion

The most important factor for achieving R0 resection for perihilar cholangiocarcinoma is margin-negative status of the remnant liver, including the proximal hepatic artery, portal vein, and bile duct. The actual surgical procedure can be performed in several ways. The THA technique first performs the partial hepatic parenchymal transection toward the right-side hilar plate for left hepatectomy or left-side hilar plate for right hepatectomy to expose the hilar plate and decide the proximal transected plane. After separating the proximal hepatic artery, portal branch, and bile duct at the transected plane, the transection of proximal bile duct is performed to confirm proximal margin-negative status under a wide surgical view.

Advanced perihilar cholangiocarcinoma easily invades major blood vessels including the hepatic artery and portal vein because of its anatomical characteristics. Hepatobiliary surgeons frequently have to judge the necessity of combined resection and reconstruction of the major blood vessels. In this situation, the most critical aspect in achieving a safe resection and reconstruction is whether or not encircling of the hepatic artery and portal branch at the future remnant side can be performed. If impossible, abandoning the curative resection should be considered. THA provides a wide surgical view and enables the surgeon to confirm the resectability and possibility of reconstruction of the major blood vessels early in the operation.

To achieve R0 resection, the absence of residual tumor of the proximal bile duct is one of the most crucial prognostic factors.^2^ Preoperative imaging studies are usually used to determine the type of hepatectomy and the cutting line of proximal bile duct. Choi et al. reported that the accuracies of CT, ERCP, and IDUS to evaluate the longitudinal extensions of perihilar cholangiocarcinoma before biliary drainage were 66.6, 60, and 90%, respectively.^15^ However, once a biliary catheter was inserted, the accuracy of IDUS in assessing longitudinal cancer extension declined to 71–72% due to bile duct wall thickening due to the inflammatory change induced by mechanical stimulation.^16^ In clinical practice, biliary catheters have been inserted in most cases of perihilar cholangiocarcinoma preoperatively. Therefore, in patients with perihilar cholangiocarcinoma who have a hilar plate with inflammatory change caused by the biliary catheter and obstructive cholangitis, and/or with tumor invasion, our THA procedure has the benefit of widely exposing the hilar plate without touching the severe inflamed and/or tumor invasion areas. It is also a useful technique to confirm the margin status of the proximal bile duct under a clear surgical view early in surgery. In patients with margin-positive hepatic proximal bile ducts, additional bile duct resection is usually performed. However, such limited resection of a margin-positive proximal bile duct did not improve survival when compared with patients in whom margin-negative proximal bile ducts was achieved at the first bile duct resection, even when a margin-negative status could be obtained with additional resections.^17^^,^ 18 In our study, two patients achieved margin-negative status upon additional hepatic proximal bile duct resections. Unfortunately, one patient died of liver metastasis at 13 months and the other of local recurrence with liver metastasis at 33 months after operation. Therefore, it is important to obtain cancer-free margins of the hepatic proximal bile duct at the first resection. With the THA procedure, margin-negative status of the residual hepatic proximal bile duct can be achieved in more than 80% of patients at the first resection, which compares favorably to those in other leading centers.^17^^,^ 18 Our THA procedure does not have an unfavorable comparison.^1^ The achievement of margin-negative status for perihilar cholangiocarcinoma might be dependent on selection of the relevant type of hepatectomy based on precise preoperative evaluation of imaging studies, but not on surgical procedure.

Margin-positive status of the proximal bile duct as a result of periductal invasion and carcinoma in situ occurs in about 10% of perihilar cholangiocarcinoma surgeries, even if additional resections are performed. In the most common surgical procedure for perihilar cholangiocarcinoma, the margin status of the proximal bile duct is usually determined at the final step of the procedure. In this situation, it is impossible to change the type of hepatectomy because it has already occurred. In THA, we confirm the margin status of the proximal bile duct as one of the first steps. When a negative margin of the proximal bile duct cannot be obtained even after additional resections, the THA procedure enables us to change the major hepatectomy to an extensive hilar bile duct resection because hepatectomy is not performed^4^^,^ 19 early in the operation. During the same study period, we tried major hepatectomy using the THA procedure in 24 patients with perihilar cholangiocarcinoma, of whom one (70-year-old female) could not have a hepatectomy performed. In this case, the planned left hepatectomy was changed to hilar bile duct resection early in the operation, because severe periductal invasions of both the anterior and posterior bile duct margin were found. The left side of the bile duct was used for the hepaticojejunostomy even though its ductal margin was positive. The patient developed anastomotic stenosis of the hepaticojejunostomy at 3 months and died at 15 months after the operation. On the other hand, two patients were intraoperatively diagnosed as HM positive. Therefore, the concept that THA enables us to change the major hepatectomy to a hilar bile duct resection early in the operation is not always applicable when the proximal bile duct margin is intraoperatively diagnosed as cancer positive, because hilar bile duct resection cannot be performed in patients whose future resected liver has severe cancerous invasion of the bile duct and hepatic vessels.

In the present study, we had to address whether neoadjuvant chemotherapy or our THA procedure influenced the negative margin rate of the bile duct. There have been few reports on the significance of preoperative or downsizing chemotherapy or chemoradiotherapy for advanced biliary tract cancer. Although the number of cases studied was small, Kato et al. concluded that preoperative chemotherapy with gemcitabine enabled the downsizing of initially unresectable locally advanced biliary tract cancer, with radical resection made possible in 8 (36.4%) of 22 patients. Four of these patients achieved R0 resection.^20^ In the present study, 2 of the 23 patients were diagnosed as cancer positive at the proposed cutting line of the intrahepatic bile duct based on preoperative biopsy. Both patients obtained negative preoperative biopsies of the bile duct at the cutting line after two cycles of chemotherapy. Finally, R0 resection could be performed in both patients. Accordingly, it is considered that preoperative chemotherapy, but not our new technique, might have some effect to obtain no cancerous proximal margin of the bile duct. However, further study is needed.

In an attempt to avoid mortality, it is important for us to perform a safe combined resection and reconstruction of the portal vein and/or hepatic artery. According to a review article for perihilar cholangiocarcinoma,^1^ portal vein resection and reconstruction (0–44%) was more frequently performed compared with hepatic artery reconstruction (0–18%). The timing of portal vein reconstruction can be classified into three periods: before, in the middle of, and after hepatic parenchymal transection. Portal vein resection and reconstruction before hepatic parenchymal transection requires performing in a relatively small surgical view. Meanwhile, the resection and reconstruction after hepatic parenchymal transection requires continuing the hepatic resection procedure with limited movement of the resection side of the liver because the bilateral sides of the liver are connected by the portal vein. Therefore, our procedure in which portal vein resection and reconstruction is performed in the middle of hepatic parenchymal transection offers a wide surgical view, which overcomes the faults of either alternative timing.

Regarding the significance of combined portal vein resection and reconstruction, three meta-analyses on combined portal vein resection for hilar cholangiocarcinoma were recently reported.^21^^–^ 23 These studies demonstrated a survival benefit of portal vein resection for hilar cholangiocarcinoma with portal vein invasion but did not recommend routine portal vein resection unless necessary. Regarding combined hepatic artery resection and reconstruction, there have been no meta-analyses because of a lack of large study cohorts. Nagino et al. retrospectively reviewed 50 patients with advanced cholangiocarcinoma who underwent hepatectomy with simultaneous portal vein resection and hepatic artery resection. They reported that R0 resection was achieved in 33 (66.0%) patients and the 5-year survival rate was 30.3%.^24^ Matsuyama et al. also reviewed 44 patients with advanced cholangiocarcinoma who underwent hepatectomy with simultaneous hepatic artery resection. They reported that R0 resection was achieved in 35 (69.5%) patients and the 5-year survival rate was 22.3%.^25^ These studies concluded that hepatic artery resection offered a survival benefit in selected patients. However, the significance of hepatic artery resection for advanced hilar cholangiocarcinoma is still controversial towing to increased morbidity and mortality without a proven survival benefit or an improvement in the rate of clear margin resections.^23^^,^ 26

THA for perihilar cholangiocarcinoma had some disadvantages. One of the disadvantages was blood loss from the cut surface of the precedent partial hepatic parenchyma resection. However, the advancement of surgical devices and tissue sealing sheets enabled us to achieve adequate hemostasis at the hepatic cut surface. Another disadvantage was bile leakage from the remnant proximal bile duct cut end early in the operation. This issue was overcome by inserting soft tubes into all hepatic proximal bile ducts to drain bile juice outside of the surgical field. Another disadvantage was starting the partial hepatic parenchymal resection without confirming the demarcation line, except in patients who underwent preoperative portal vein embolization. Intraoperative US helps us to determine the hepatic resection line along the line of the preserved major hepatic vein, but we experienced some patients who developed ischemia along the cut margin of the residual liver which required additional hepatic resection. This study itself has a few limitations. Additionally, the patient cohort was small and the observation period was short. Further evaluation is needed to justify the use of this technique.

## Conclusion

The THA procedure for perihilar cholangiocarcinoma is considered useful and practicable regardless of local tumor extension, enabling surgeons to determine tumor resectability and perform safe resection and reconstruction of the major blood vessels early in the operation under a wide surgical view.
